# Ambulance officers' use of online clinical evidence

**DOI:** 10.1186/1472-6947-6-31

**Published:** 2006-07-27

**Authors:** Johanna I Westbrook, Mary T Westbrook, A Sophie Gosling

**Affiliations:** 1 Centre for Health Informatics, University of New South Wales, Kensington 2052, NSW, Australia; 2 Centre for Clinical Governance Research in Health, University of New South Wales, Kensington 2052, NSW, Australia; 3 Department of Psychology*, Royal Holloway, University of London, Egham Hill, Egham, Surrey, UK

## Abstract

**Background:**

Hospital-based clinicians have been shown to use and attain benefits from online evidence systems. To our knowledge there have been no studies investigating whether and how ambulance officers use online evidence systems if provided. We surveyed ambulance officers to examine their knowledge and use of the Clinical Information Access Program (CIAP), an online evidence system providing 24-hour access to information to support evidence-based practice.

**Methods:**

A questionnaire was completed by 278 ambulance officers in New South Wales, Australia. Comparisons were made between those who used CIAP and officers who had heard of, but not used CIAP.

**Results:**

Half the sample (48.6%) knew of, and 28.8% had used CIAP. Users were more likely to have heard of CIAP from a CIAP representative/presentation, non-users from written information. Compared to ambulance officers who had heard of but had not used CIAP, users were more likely to report better computer skills and that their supervisors regarded use of CIAP as a legitimate part of ambulance officers' clinical role. The main reasons for non-use were lack of access(49.0%) and training(31.4%). Of users, 51.3% rated their skills at finding information as good/very good, 67.5% found the information sought all/most of the time, 87.3% believed CIAP had the potential to improve patient care and 28.2% had directly experienced this. Most access to CIAP occurred at home. The databases frequently accessed were MIMS (A medicines information database) (73.8%) and MEDLINE(67.5%). The major journals accessed were Journal of Emergency Nursing(37.5%), American Journal of Medicine(30.0%) and JAMA(27.5%).

**Conclusion:**

Over half of ambulance officers had not heard of CIAP. The proportion who knew about and used CIAP was also low. Reasons for this appear to be a work culture not convinced of CIAP's relevance to pre-hospital patient care and lack of access to CIAP at work. Ambulance officers who used CIAP accessed it primarily from home and valued it highly. Lack of access to CIAP at central work locations deprives ambulance officers of many of the benefits of an online evidence system.

## Background

Health care managers seeking to implement organisational and system-wide information system interventions to support improvements in clinical practice have a wide selection of possibilities from which to choose. However when selection criteria include evidence of intervention effectiveness the options rapidly fall away. We have undertaken a program of research focused upon examining the effectiveness of online evidence systems to support improved clinical decision-making and patient care. This research has centred on examination of the Clinical Information Access Program (CIAP) [1], an online evidence website implemented by the New South Wales Health Department in 1997. CIAP provides around 55,000 health practitioners in the state's public hospital sector with access to clinical databases and journals 24 hours a day, both at point-of-care and at home. It is the largest health evidence service undertaken in Australia and internationally.

Several studies of hospital clinicians' use of CIAP have demonstrated that this intervention is effective in supporting clinicians in their provision of clinical care [2], has influenced their information-seeking behaviours [3]and increases the accuracy of clinical decision-making [4]. A state-wide survey of 5,511 clinicians revealed significant differences in the experiences and use of CIAP reported by doctors, nurses and allied health professionals [5-7]. When administering this survey we took the opportunity to survey ambulance officers whose main work occurs outside hospitals but who have regular contact with hospitals. Ambulance officers all have passwords to access the online evidence service, but there was considerable variation in the extent to which ambulance stations had terminals available from which staff could access CIAP.

While there is an enormous literature relating to evidence-based practice in the medical, nursing and allied health professions, there is very limited data relating to the extent to which ambulance professionals have adopted, or been supported in their use of, evidence-based practice. To our knowledge no previous studies of pre-hospital professionals' use of online evidence systems have been published. Using a convenience sample of ambulance officers we aimed to examine their patterns utilisation of the CIAP, and to compare the characteristics of ambulance officers who used CIAP with those officers who knew of, but had not used CIAP.

## Methods

### Questionnaire

**Figure 1:**
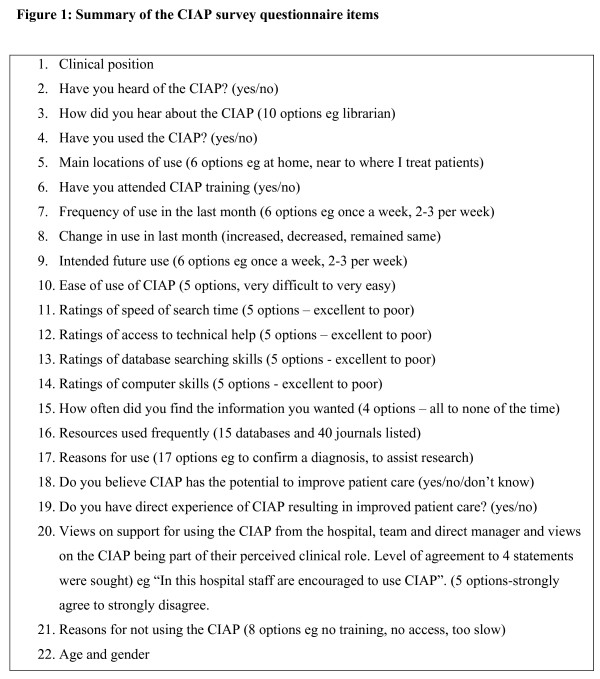
**Summary of the CIAP survey questionnaire items**. 1. Clinical position. 2. Have you heard of the CIAP? (yes/no). 3. How did you hear about the CIAP (10 options eg librarian). 4. Have you used the CIAP? (yes/no). 5. Main locations of use (6 options eg at home, near to where I treat patients). 6. Have you attended CIAP training (yes/no). 7. Frequency of use in the last month (6 options eg once a week, 2–3 per week). 8. Change in use in last month (increased, decreased, remained same). 9. Intended future use (6 options eg once a week, 2–3 per week). 10. Ease of use of CIAP (5 options, very difficult to very easy). 11. Ratings of speed of search time (5 options – excellent to poor). 12. Ratings of access to technical help (5 options – excellent to poor). 13. Ratings of database searching skills (5 options – excellent to poor). 14. Ratings of computer skills (5 options – excellent to poor). 15. How often did you find the information you wanted (4 options – all to none of the time). 16. Resources used frequently (15 databases and 40 journals listed). 17. Reasons for use (17 options eg to confirm a diagnosis, to assist research). 18. Do you believe CIAP has the potential to improve patient care (yes/no/don't know). 19. Do you have direct experience of CIAP resulting in improved patient care? (yes/no). 20. Views on support for using the CIAP from the hospital, team and direct manager and views on the CIAP being part of their perceived clinical role. Level of agreement to 4 statements were sought) eg "In this hospital staff are encouraged to use CIAP". (5 options-strongly agree to strongly disagree. 21. Reasons for not using the CIAP (8 options eg no training, no access, too slow). 22. Age and gender.

A 22 item paper questionnaire (Figure 1) was developed to investigate health professionals' knowledge and use of CIAP. The survey was designed principally to obtain information regarding a) how many health professionals had heard of the online evidence system, b) if and c) how frequently they used it, d) their reasons for not using the system and e) whether they believed the use of the system had impacted upon patient care. The questionnaire, comprised close-ended questions, multiple response items and attitudinal scales (Figure 1). Potential reasons for use of CIAP included in the questionnaire were adapted from a US study of clinicians' use of MEDLINE [8]. The survey was pilot tested and minor modifications were made to the wording of some questions to increase clarity [5]. The study was approved by the University of NSW's Human Research Ethics Committee. Consent was given by completing and returning the survey questionnaire.

### Procedure

The survey was conducted in 2001 in 65 randomly selected state hospitals where a survey coordinator ensured that 25% of the medical, nursing and allied health staff completed the paper-based questionnaire [5]. Although the questionnaire was developed to investigate the experiences of hospital-based health professionals, the NSW Ambulance Service requested that ambulance officers be included in the survey as much of the questionnaire was considered pertinent to its workforce. The Service requested 2,000 questionnaires which were then sent by the Ambulance service to Ambulance Stations throughout the state for distribution to qualified ambulance officers. The researchers had no control over and were not involved in the processes of distribution or collection of the surveys which may have been less than optimal. Of these 2000 surveys 278 were completed, a return rate of 14%. Chi square analyses and a t-test were used to compare the characteristics of ambulance officers who were CIAP users with those officers who knew of, but had not used, CIAP. Some respondents (ranging from 1–5 in number) failed to answer a few questions. Percentages were calculated in terms of the number answering each item. Some questionnaire items were not relevant to ambulance officers working outside hospitals and were omitted from the analysis.

### Sample

The convenience sample of 278 ambulance officers had a mean age of 38.0 years (SD = 9.1 years, range = 21–68 years); 72.6% were males and 27.4% were females. The gender distribution of the sample was similar to that of the state's ambulance staff [9]. Respondents (N = 135) who had heard about CIAP were the primary focus of the study. They did not differ significantly in age from officers who had not heard of CIAP (Heard of CIAP: mean = 37.3 years, SD 9.39; Not heard: mean = 37.3 years, SD = 10.54, t = -0.008, df 267, p = 0.994). Those who had heard of CIAP did not differ in gender from officers who had not heard of CIAP (respectively 75.4% males compared to 69.8% of officers who had not heard of CIAP, χ^2 ^= 1.034, df 1, p = 1.034).

## Results

### Knowledge of CIAP and characteristics of users and non-users

Respondents were asked "*Have you heard of CIAP?*" and "*Have you used CIAP?*" Of the 278 ambulance officers almost half knew of, and over a quarter had used CIAP (Table 1). Of those who knew of CIAP, 59.3% had used it but less than a quarter of users had attended a training course in CIAP. The gender of users and non-users was similar (χ^2 ^= 0.15, df 1, p = 0.696) as were the average ages of users and non-users (respectively mean = 38.0 years and 36.8 years; t = 0.78, df 126, p = 0.44).

**Table 1:** Ambulance officers' knowledge and use of CIAP

***Total sample of ambulance officers (n = 278)***	
Heard of CIAP	135 (48.6%)
Used CIAP	80 (28.8%)
	
***Ambulance officers who had heard of CIAP (n = 135)***	
Used CIAP	80 (59.3%)
	
***Ambulance officers who used CIAP (n = 80)***	
Attended CIAP training course	18 (22.5%)

We investigated whether responses to the survey differentiated officers who used CIAP from those who had heard of, but not used it. These analyses (Table 2) revealed that users rated their computer skills more highly than did non-users. Users first heard of CIAP from different sources than did non-users. The most frequent source of knowledge for both groups was a work colleague but users were more likely to have received information from a CIAP representative, a presentation on CIAP, a librarian or at university. Non-users were much more likely to have learnt of CIAP from written information. Users were significantly more likely to agree with the statements *"I think CIAP is a legitimate part of my clinical role" *and *"My direct supervisor thinks that using CIAP is a legitimate part of my clinical role"*. However users and non-users equally disagreed (43.1%,) or were undecided (34.3%) that *"People I work with use CIAP as a means of obtaining up-to-date clinical information" *(χ^2 ^= 4.21, df 2, p = 0.122).

**Table 2:** Results of chi square analyses comparing CIAP users and non-users who had heard of CIAP

**Questions and responses**	**CIAP users**	**CIAP non-users**	**Total**
***Please rate your computer skills***			
Poor/fair	16 (20.2%)	25 (49.0%)	41 (31.5%)
Good	28 (35.4%)	18 (35.3%)	46 (35.4%)
Very good/excellent	35 (44.3%)	8 (15.7%)	43 (33.1%)
χ^2 ^= 15.80, df 2, p = 0.000			
***How did you first hear about CIAP?***			
CIAP representative/presentation	13 (16.4%)	1 (2.0%)	14 (10.8%)
Written information	7 (8.9%)	16 (31.4%)	23 (17.7%)
Work colleague	32 (40.5%)	19 (37.2%)	51 (39.2%)
Staff orientation	10 (12.7%)	7 (13.7%)	17 (13.1%)
Librarian/university	7 (8.9%)	0 (0.0%)	7 (5.4%)
Other	10 (12.7%)	8 (15.7%)	18 (13.8%)
χ^2 ^= 19.76, df 5, p = 0.001			
***Indicate your support for: I think using CIAP is a legitimate part of my clinical role***			
Strongly disagree/disagree	3 (4.1%)	7 (15.9%)	10 (8.5%)
Undecided	13 (17.8%)	19 (43.2%)	32 (27.3%)
Agree/strongly agree	57 (78.1%)	18 (40.9%)	75 (64.1%)
χ^2 ^= 16.85, df 2, p = 0.000			
***Indicate your support for: My direct supervisor thinks that using CIAP is a legitimate part of my clinical role***			
Strongly disagree/disagree	21 (30.0%)	12 (27.3%)	33 (28.9%)
Undecided	18 (25.7%)	23 (52.3%)	41 (36.0%)
Agree/strongly agree	31 (44.3%)	9 (20.4%)	40 (35.1%)
χ^2 ^= 9.74, df 2, p = 0.008			

### Reasons for using or not using CIAP

Ambulance officers who used CIAP were asked to check as many of 17 reasons for using CIAP as applied to them. The reasons most frequently given were to: fill a knowledge gap (77.5%), undertake personal education eg for a specific course or project (71.3%), gain access to a standard reference (50%), assist research (48.8%), support the education of others (33.8%), improve patient outcomes (15.0%), confirm a clinical decision (12.5%), settle a dispute or controversy regarding diagnosis or treatment (11.3%), make a diagnosis (5.0%), develop a treatment plan (5.0%) and review policies or guidelines (5.0%).

When officers who knew of but had not used CIAP, were asked to check their reason(s) for non-use from a set of options their responses were: no access (49.0%), no training (31.4%), lack of time (17.6%), use other information sources (15.7%), don't need information from CIAP to do my job (9.8%), difficult to use (3.0%), too slow (2.0%) and other (19.6%).

### Frequency of use of CIAP

CIAP users were asked, "*In the last month, how often have you used CIAP?*" The most frequent response was never (30.0%), followed by once (26.3%), 2–3 times (23.8%), 2–6 times per week (13.8%), once a week (6.3%), and daily 0.0%. When asked their intentions regarding their future use 1.3% said they did not plan to use CIAP, 17.7% planned to use it less than once a month, 45.6% checked 1–3 times per month, 16.5% said once per week, 17.7% said 2–6 times per week and 1.3% said daily. Thus users intended to increase their usage in the future; currently 20.1% of users were accessing CIAP once a week or more while in the future 35.5% planned to do so.

### Ease of using CIAP

Users were asked to rate CIAP in terms of how easy it was to use. It was assessed as very easy by 5.1%, easy by 26.6%, neither easy nor difficult by 48.1%, difficult by 17.7% and very difficult by 2.5% of users. When requested to rate the speed of search time on CIAP 2.5% said excellent, 21.5% very good, 53.2% good, 21.5% fair and 1.3% poor. Users were asked to rate their access to technical help regarding CIAP. Only 1.3% said access to help was excellent, 8.0% checked very good, 45.3% good, 41.3% fair and 4.0% poor. Users rated their "*skills in using CIAP to find information*". No one said excellent, 11.3% checked very good, 40.0% good, 40.0% fair and 8.8% poor.

### Location of access to CIAP

The questionnaire asked respondents the locations where they accessed CIAP. These questions were aimed at CIAP use by clinicians in hospitals rather than ambulance officers in the field. Thus only 2.5% of ambulance officers had access to CIAP "*near to where you treat/manage patients*", 6.3% had access in a university library, 3.8% in a hospital library, 30.0% in an "*office away from the clinical area*", 83.8% had access at home, and 11.3% in other places. Most ambulance officers (66.3%) only mentioned one place where they had access, 30.0% had access in two locations and 3.8% in three locations.

Respondents were asked to estimate the percentage of time they used CIAP in each of four locations; home, where you treat patients, other places in the hospital and other. The percentages were to total 100%. The most frequent place where ambulance officers used CIAP was at home; 65 of the 80 CIAP users said they did so and they estimated on average that 80.6% of their usage occurred there. Only 5 officers used CIAP near point-of-care and they estimated that an average of 51.0% of their usage occurred there. Fifteen officers used CIAP in "*other places in the hospital*" and on average 49.0% of their access time was spent there. Nineteen officers said they used CIAP in other places and on average 66.8% of their usage occurred there. Thus most users accessed CIAP at home and most of their use of CIAP occurred there.

### Databases accessed

At the time of the survey CIAP provided access to 15 databases. Ambulance officers who used CIAP were asked to indicate from a list the databases they used most frequently. These were MIMS (a medicines information database) (73.8%), MEDLINE (67.5%), Harrison's Book of Internal Medicine (33.8%), Interactive ECG Tutorials (28.8%), Cochrane (21.3%), CINAHL (17.5%), EMBASE (11.3%), Australian Medicines Handbook (10.0%), Therapeutic Guidelines (8.8%), Medweaver (7.5%), Micromedex (6.3%), Medix case-based learning (6.3%), the Medical Officer's Handbook (5.0%), PsychINFO (3.8%) and Interactive WWW internet tutorials (2.5%). The average number of databases checked was 3.0 (SD 1.7, range 0–8). Only two CIAP users did not frequently access any databases

### Journals accessed

There were 16 full-text medical, 14 nursing and 10 mental health journals available to CIAP users at the time of the survey. Respondents were asked to indicate from a list which they used most frequently. These were the Journal of Emergency Nursing (37.5%), American Journal of Medicine (30.0%), Journal of the American Medical Association (27.5%), British Medical Journal (22.5%), New England Journal of Medicine (20.0%), The Lancet (16.3%), Advances in Nursing Science (10.0%), Journal of Clinical Nursing (10.0%), Annals of Internal Medicine (8.8%), Journal of Clinical Investigation (7.5%), Advances in Nursing Science (7.5%), Nursing Standard (7.5%) and Pediatrics (7.5%). The average number of frequently referred to journals was 2.6 per user (SD 2.3, range 0–11). Thirty percent of CIAP users did not list any journal as frequently used.

### Effectiveness of CIAP

Respondents were asked, *"Considering the times you have used CIAP, how often did you find the information you wanted?" *Thirteen percent said all of the time, 54.5% reported most of the time, 31.3% some of the time and no one checked never. Users were asked, *"Do you believe CIAP has the potential to improve patient care?"*. Most (87.3%) answered yes, 11.4% said they did not know and 1.3% responded no. When asked *"Do you have direct experience of CIAP resulting in improved patient care?*" 28.2% of users said yes and 71.8% replied no.

## Discussion

Ambulance officers' questionnaire responses revealed lower levels of knowledge of CIAP (48.6%) than did other health professions surveyed at the same time; 58% of nurses [5], 72% of doctors [7] and 82% of allied health staff [10] had heard of CIAP. Despite indicators of ambulance officers' favourable endorsement of practice improvement initiatives such as CIAP [1] the proportion of ambulance officers who knew of and had also used CIAP (59.3%) was substantially less than for other health professions; 74% of nurses [5], 84% of doctors [7] and 76% of allied health [10] knew about and used CIAP. Of the total sample of ambulance officers 5.0% had heard of CIAP from a CIAP representative or at a presentation compared to 6.4% of all nurses and 6.1% of doctors. Thus lack of exposure to these informational inputs via a CIAP representative or presentation, does not appear to be a major factor in explaining ambulance officers' poorer knowledge of CIAP.

Ambulance officers' reasons for non-use differed from those of other professions. Nearly half of the ambulance officers who were non-users indicated that the reason for this was that they did not have access to CIAP compared to, for example, 22.3% of nurse non-users. While 63.5% of nurses cited lack of CIAP training as a reason for non-use, only 31.4% of ambulance officers did so. Most nurse users accessed CIAP near point-of-care (58.2% vs. 2.5% of ambulance officers), in the hospital library (31.9% vs. 3.8% of ambulance officers) or in an office away from the clinical area (29.7% vs. 30.0%). Most ambulance officers accessed CIAP at home (83.8%) compared to 41.7% of nurses [5]. Thus lack of access to CIAP would seem to be a major contributing factor to lower use of CIAP by ambulance professionals. Few resources available on CIAP at the time of the survey were targeted at pre-hospital emergency care and this is also likely to have reduced the relevance of CIAP. This was similarly a problem for allied health professional groups and contributed to some groups' lower rates of CIAP use [10]. Since the survey CIAP has substantially increased the resources available with several 100 fulltext journals available. However, as Tippett et al [11] point out there remains a significant shortage of research in pre-hospital emergency care limiting the availability of high quality evidence to guide evidence-based practice.

A further factor contributing to lower use among ambulance officers appears be the lack of support and encouragement to use CIAP in their work environments. Comparison of nurses' and ambulance officers' responses to statements about the legitimacy of using CIAP as part of one's clinical role showed stronger support for this proposition among nurses of whom 71.9% agreed/strongly agreed compared to 64.1% of ambulance officers. The difference between nurses and ambulance officers was much greater regarding agreement with the proposition that their direct supervisor saw using CIAP as a legitimate part of their role; 50.4% of nurses agreed/strongly agreed compared to 35.1% of ambulance officers. In response to the statement "*People I work with use CIAP as a means of obtaining up-to-date information*" only 22.5% of ambulance officers agreed/strongly agreed, while 53.8% of nurses did so. Thus the work cultures of nurses and other health professional groups were more supportive of the use of CIAP. This, as well as difficulties of access, may account for ambulance officers' much greater usage of CIAP at home.

Those ambulance officers who used CIAP were clearly committed to its use as they devoted out-of- work time to this activity. Not only did more ambulance professionals access CIAP at home than did any other professional group, but they also undertook most of their work with CIAP there. The smaller proportion of nurse users who used CIAP at home claimed that they performed on average 51.4% of their work with CIAP there, compared to ambulance officers who did 80.6% of their CIAP work there. Of ambulance officers who used CIAP, 67.5% reported finding information they sought on CIAP all or most of the time compared to 64.0% of nurses. Ambulance professionals' ratings of the speed of CIAP were similar to those of nurses, but their ratings of ease of use of CIAP and their skills in CIAP use were slightly lower than were nurses' ratings. Nurse users accessed CIAP more frequently (26.6% had used CIAP in the last week) than ambulance officers (20.1%). Most ambulance officers (87.3%) and nurses (84.2%) who used CIAP considered that CIAP had the potential to improve patient care, though fewer ambulance officers (28.2%) than nurses (35.1%) reported direct evidence of this. Lack of access to CIAP at point-of-care was a major barrier to ambulance professionals being able to utilise the full benefits of CIAP. The survey shows that compared to other health professions there was less cultural support for ambulance officers to use CIAP. Nevertheless a committed group of officers, who primarily used CIAP away from work, reported experiences of the benefits of CIAP similar to those of user groups in other professions.

A limitation of this study is that the sample represented a smaller and probably less representative proportion of ambulance professionals than was the case with the other health professions surveyed. We had limited control over the survey distribution and have no information about non-responders. It is possible that CIAP users were more likely to complete the survey than non-users. However the fact that over half of the respondents reported not having heard of CIAP provides some reassurance that this group was not significantly under-represented. Also the demographics of those who had heard of CIAP did not differ significantly from those who had not heard of CIAP. Given the lack of research undertaken about this group of health professionals we believe the results provide an important glimpse into the profession's responsiveness to adopting evidence-based practice measures where possible. Ambulance officers have been shown to hold highly favourable attitudes toward other health improvement initiatives such as the NSW Safety Improvement Program [12]. Early assessments of the CIAP provided some evidence that ambulance officers were receptive to the benefits that CIAP might provide. In her report of an on-line survey of CIAP Ayres [1]quoted the comments of an ambulance officer: *"The CIAP results in greater clinical knowledge, so thanks for a great resource. This is absolutely fabulous for ambulance officers. We are often left out in terms of on-going education, and access to this site enables us to keep up with current trends, research and medications." *(p181) This limited evidence all suggests that ambulance officers are open to initiatives aimed at improving care processes and the adoption of evidence-based practices.

## Conclusion

We now have a growing body of research which demonstrates that access to online evidence systems improves professionals' accuracy of [4,13,14], and confidence in [15] answers to clinical questions, and positively impacts upon care delivery and patient outcomes [2,16,17]. The results of this survey suggest that ambulance officers as a professional group are also likely to reap these benefits when given easy access to, and encouragement by their work culture to use, such resources. Evidence-based health policy makers and organisational leaders should work towards increasing access to online evidence systems for health professionals both within and outside hospitals.

## Competing interests

The author(s) declare that they have no competing interests.

## Authors' contributions

JW and ASG designed and conducted the survey. JW and MW undertook the analysis and prepared the first draft of the manuscript. All authors reviewed and refined the manuscript. All authors read and approved the final manuscript.

## Pre-publication history

The pre-publication history for this paper can be accessed here:


